# An Intraoperative Technique to Assess Tissue Tension and Leg Length When Aligning the Hip Centre of Rotation With the Acetabulum in Hip Arthroplasties

**DOI:** 10.7759/cureus.65860

**Published:** 2024-07-31

**Authors:** Zaid Al Ani, Khalid Sharif, Sumant C Verghese, Sarvpreet Singh, Vijay V Killampalli

**Affiliations:** 1 Trauma and Orthopaedics, Peterborough City Hospital, Peterborough, GBR; 2 Trauma & Orthopaedics, Diana, Princess of Wales Hospital, Grimsby, GBR; 3 Trauma & Orthopaedics, Hinchingbrooke & Peterborough City Hospital, North-West Anglia NHS Foundation Trust, Huntingdon, GBR; 4 Lower Limb Arthroplasties, Peterborough City Hospital, Peterborough, GBR; 5 Trauma and Orthopaedics, North-West Anglia NHS Foundation Trust, Huntingdon, GBR

**Keywords:** centre of rotation, hip offset, soft tissue tension, leg length inequality, hip and knee replacement

## Abstract

Hip arthroplasties are cost-effective procedures; however, instability and leg length discrepancy are common complications that can lead to higher revision rates and patient dissatisfaction. Preoperative planning aids surgeons in choosing the right offset and neck length before surgery. Nonetheless, intraoperative measures are still necessary due to the differences dictated by the surgical procedure. Several hip trials might be needed to reach the optimum choice of implants.

We have introduced a technique that utilizes the trunnion as a reference point to the hip centre of rotation, matching it with the acetabulum centre of rotation after applying the necessary soft tissue tension. This serves as a proximal reference point. Using the trunnion, as opposed to the trial head, allows for a better assessment of tissue tension within the acetabular void, avoiding constraints imposed by the applied trial head. Additionally, determining the acetabulum's centre of rotation is challenging if obscured by the trial head. Matching the two tibial tuberosities indicates the correct leg length, serving as the distal reference point. Both reference points should be considered together to select the right neck length and offset for optimal tissue tension.

This technique has been tested on hip arthroplasty patients over five years. All hip surgeons who used this technique agree that it gives a better representation of the tissue tension, easing the challenges when preparing the acetabulum as well as reducing the need for multiple trials.

## Introduction

Total hip arthroplasty (THA) is a successful surgical intervention aimed at restoring the hip joint's function and relieving pain [1]. Accurately restoring the hip centre of rotation (HCOR) is crucial for optimizing hip biomechanics and improving the longevity of the prosthesis [2]. However, achieving this intraoperatively poses challenges due to variations in landmarks compared to preoperative planning. Shifts in HCOR, particularly mediolateral or super inferior displacement, can lead to various adverse consequences such as leg length inequality, abnormal gait patterns, accelerated wear of bearing surfaces, component loosening, and dislocation [2-4]. These complications underscore the importance of precise HCOR restoration during THA.

Preoperative planning still plays an important role in estimating implant size, position, and orientation based on the patient's anatomy. However, it is not without limitations, as inaccuracies in predicting implant size can occur, potentially leading to implant mismatch and instability [5]. Soft tissue tensioning, particularly of the abductor mechanism, is essential for ensuring dynamic stability of the hip joint post-THA. Dysfunction or imbalance of the abductor mechanism can result in complications such as dislocation, limping, and early implant failure. Assessing soft tissue tension intraoperatively is challenging but crucial for optimizing functional outcomes and implant longevity [6].

## Technical report

**Figure 1:**
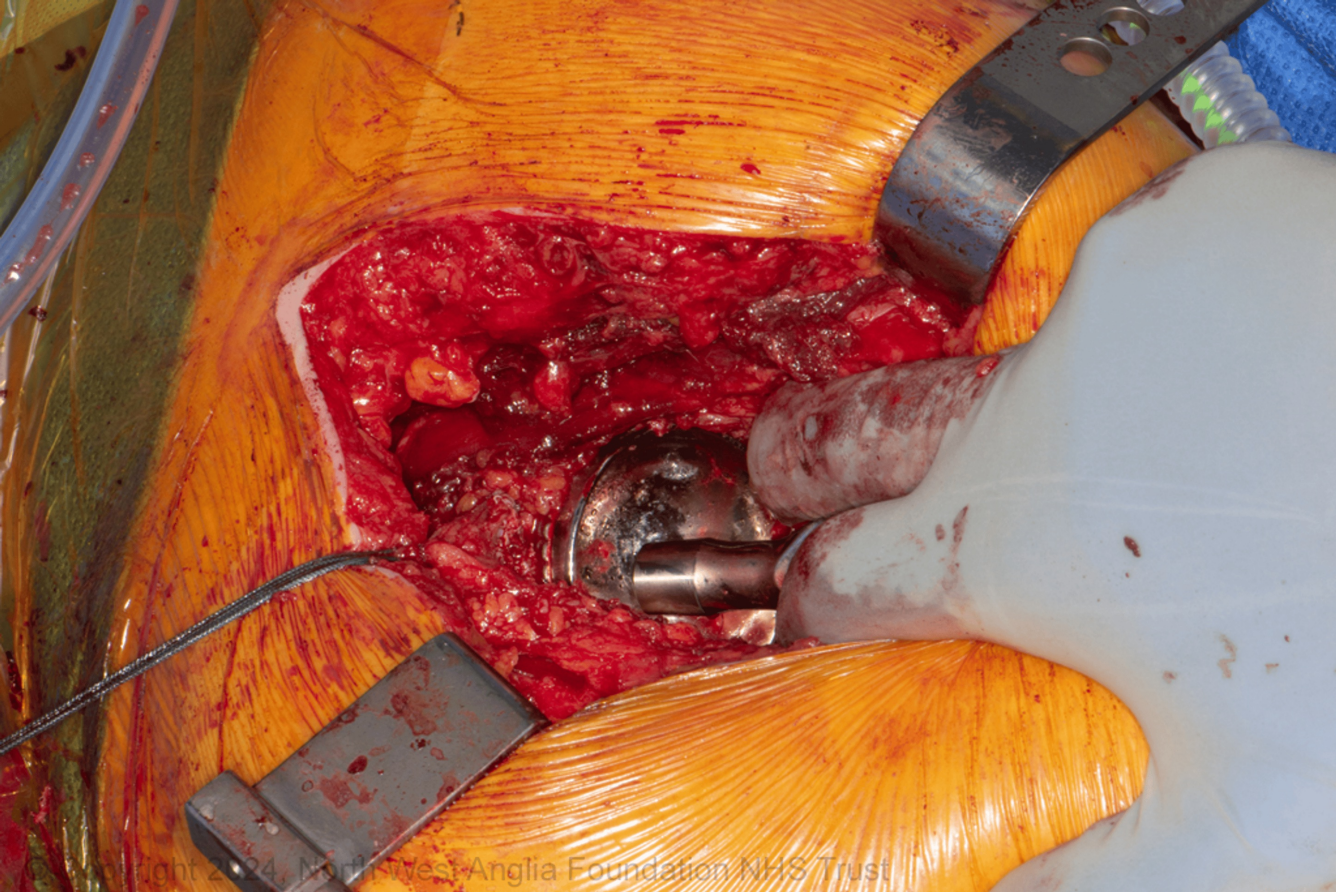
Applying tension while aligning the two centres of rotation

**Figure 2:**
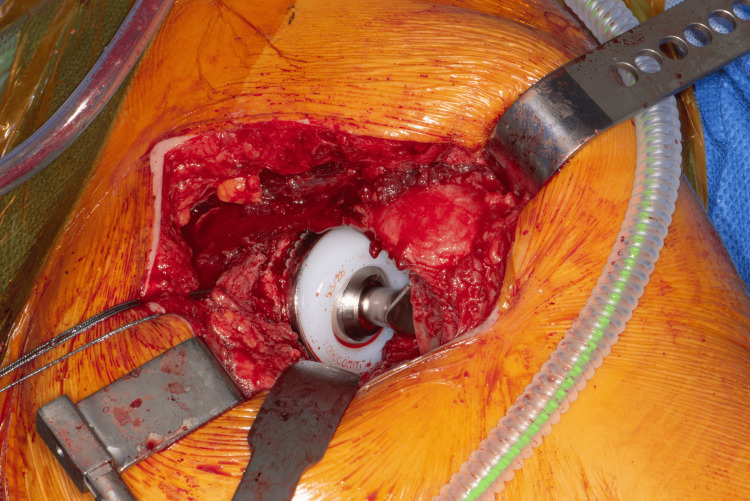
Applying the final components of THR based on tissue tension THR: total hip replacement

Preoperative planning is still recommended for patients needing hip arthroplasty. The HCOR can still be assessed on normal pelvic radiographs [7,8]. This helps identify the height of the neck cut. The patient will be positioned laterally, using lateral and front supports secured in place. The pelvis also needs to be squared to minimize possible errors when comparing the two tibial tuberosities for the distal reference point. Both posterolateral and anterolateral hip approaches can be used with this technique; good exposure is recommended to ensure good visibility (Figures 1, 2). Anatomical placement of the acetabulum cup must be implemented when possible. This re-establishes the natural biomechanics of the hip [9-11].

**Figure 3:**
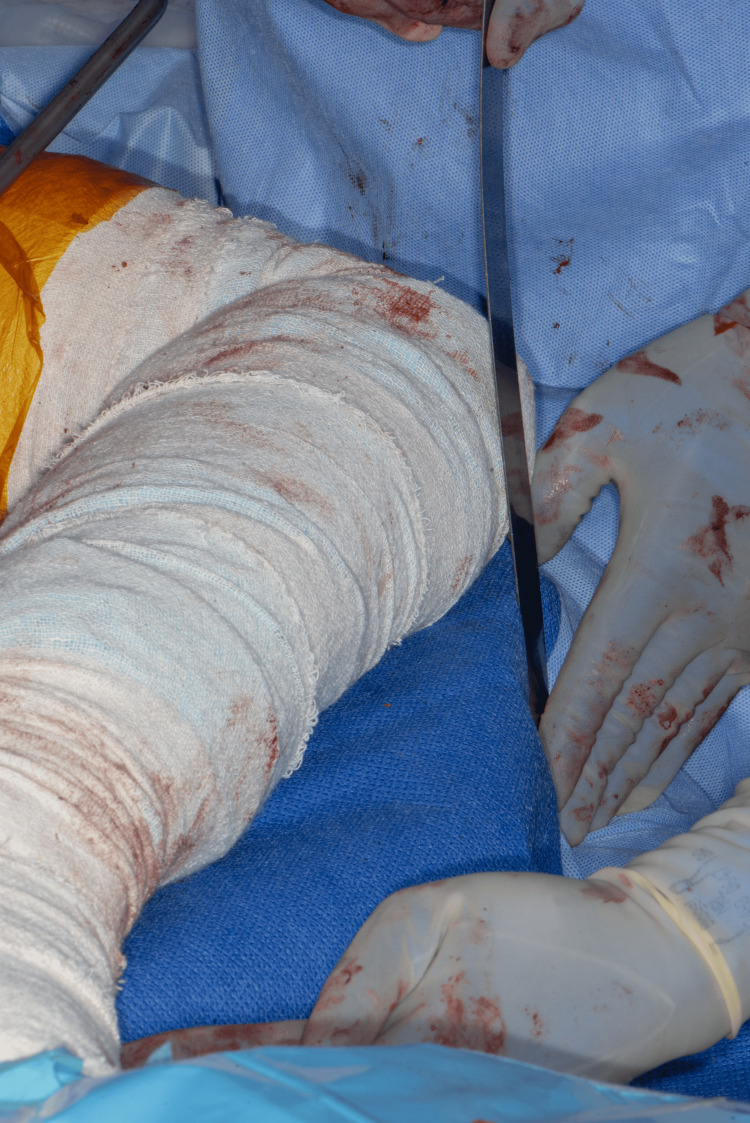
Checking the leg length by comparing the two tibial tuberosities

The upper femur is prepared using standard reamers and rasps. When the appropriate rasp is in place, the suggested modular neck is applied, and the centre of the trial trunnion now represents the new HCOR. The operating surgeon positions the trunnion in the acetabulum with the necessary tension to align the centre of the trunnion with the acetabulum's centre of rotation. Care is needed to avoid scratching the liner (a trial liner can be considered). Using a hook facilitates this manoeuvre, especially in high BMI patients. Applying the necessary tension laterally (lateral shift) to check for the correct offset is essential. If the trunnion moves laterally away from the centre of the acetabulum easily, a higher offset is needed. Then, the neck length is checked distally. Moving the trunnion within the acetabular void gives a better assessment of tissue tension, providing the surgeon with the flexibility to check the tissue tension while aligning the HCOR within the acetabulum (Figure 1). The surgeon can also check the two tibial tuberosities for leg length at the same time (Figure 3). This allows for necessary changes to the offset or neck length to address any malalignment or excessive tension before selecting the final implants (Figure 2).

This technique has been tested on all patients admitted for hemiarthroplasty as well as total hip replacement (THR) in our centre over 5 years. During this time, the operating surgeon implemented the preoperative planning measures and used tissue tension to align the HCOR with the acetabulum centre before the final trial. All surgeons agreed that this approach reduced the need for multiple trials and decreased the chance of complications in their operations.

## Discussion

Numerous authors have explored various techniques to mitigate leg length discrepancy after total hip replacement, yet no single method has proven superior. The primary challenge lies in correcting leg length without compromising stability [12]. Several strategies have been employed, including preoperative planning, intraoperative markers or imaging, complex measurements, navigation and robotic surgery.

There is a consensus among authors that absolute equalization of limb length is difficult to achieve [13]. Utilizing multiple techniques tends to produce more reliable outcomes [14]. Preoperative planning alone cannot guarantee a successful outcome [15]. Intraoperative techniques are generally more reliable and cost-effective [14]. Over 20 intraoperative techniques have been documented in the literature, most of which involve measuring a linear distance between two points: one fixed to the pelvis and the other to the femur. Ranawat et al. suggested that positioning the first point closer to the centre of rotation minimizes miscalculations caused by limb positioning. His technique involves using a vertical Steinmann pin at the infracotyloid groove of the acetabulum. Despite good outcomes, it has become less popular due to the difficulty associated with positioning the pin in that groove; this is caused by osteophytes on the posterior lip of the acetabulum [12].

Manual testing of tissue tension is a well-established procedure in total knee replacement, proving effective in achieving good outcomes when performed by experienced surgeons [16]. Similarly, the proposed technique for hip arthroplasty involves adjusting tension to match the centre of rotation, showing promise in achieving comparable results. Using the centre of rotation as a marker also minimizes the impact of limb position. This procedure allows a skilled surgeon to make necessary adjustments to the stem and neck height and offset based on tissue tension while matching the trial trunnion followed by the real trunnion to select the accurate hip components. It is non-invasive and cost-effective. This technique still requires the necessary skills to achieve good exposure to provide the necessary visibility. Positioning the trunnion within the acetabulum and applying the necessary tension can be slightly difficult in high BMI patients although using the right instruments can overcome these difficulties. 

The proposed technique also includes applying the distal two tibial tuberosities as second reference points to increase consistency; however, pelvic squaring is essential to ensure efficacy. Multiple trials with excessive force during hip reduction can affect positioning, reducing reliability. This method has been successfully applied to all patients undergoing hemiarthroplasties and total hip replacements in our hospital over five years, reducing the need for repeated trials and offering a more accurate selection of implant components. This technique has minimized tissue damage, reduced the risk of periprosthetic fractures, and improved overall surgical outcomes.

High-tech innovations have emerged, offering more precise solutions. Notable advancements include sensor-equipped modular femoral heads that measure forces within the hip joint during surgery, aiding surgeons in optimizing soft tissue tension and leg length adjustments in real time [17]. Additionally, robotic-assisted surgery has enhanced the precision of bone cuts and implant placement, potentially leading to improved alignment, leg length symmetry, and soft tissue tension [18].

However, these advanced technologies have significant drawbacks. The high costs of the acquisition and maintenance of robotic systems and sensor technologies present substantial barriers for many healthcare facilities, especially those in resource-limited settings [19]. Moreover, the expenses associated with training surgeons and operating room staff to effectively use these technologies are considerable and time-consuming. Overreliance on these technologies might diminish surgeons' proficiency in traditional techniques, which could be problematic if the technology fails or is unavailable [20]. The setup and calibration of robotic systems and other advanced devices can extend the duration of surgical procedures and increase the potential for intraoperative complications [20]. Furthermore, these systems can be susceptible to technical malfunctions, potentially leading to significant delays or necessitating a switch to conventional techniques [20].

Despite these issues, some studies indicate that advanced technology-dependent techniques can enhance radiological outcomes, particularly with robotic systems. However, the evidence regarding their impact on functional outcomes compared to conventional methods remains mixed. This variability challenges the justification of the high costs and complexity associated with these technologies [19].

While advancements such as computer-assisted navigation and intraoperative sensors offer more accurate and quantitative evaluations of soft tissue tension [18], the experience and skill of the surgeon remain crucial in achieving optimal outcomes during THA [4].

## Conclusions

Assessing soft tissue tension while levelling the trunnion with the centre of the acetabulum in hip arthroplasty provides a more reliable technique to restore hip function, reduces the need for multiple trials, and elevates patient position artefact. It is also less invasive and cost-effective. It can be an alternative to high-cost advanced technology methods in the hands of skilled orthopaedic surgeons.
